# Chicago Veterinary College—Commencement

**Published:** 1892-04

**Authors:** 


### CHICAGO VETERINARY COLLEGE—COMMENCEMENT.

The commencement exercises of the Chicago Veterinary College were held on Thursday, March 24, at 2.30 o'clock, at Hooley's Theatre, Chicago.

Diplomas were awarded to the following gentlemen: C. H. Anthony, W. S. Arbuthnot, J. G. Atchison, C. H. Bagnall, B. Baldwin, F. H. Barr, D. E. Baughman, S. H. Bauman, J. H. Beadle, A. E. Behnke, G. Brauchle, F. Briggs, F. F. Brown, M. H. Browning, L. Campbell, E. N. Chute, J. B. Clancy, W. S. Clark, P. D. Coffey, T. D. Collins, G. D. Crossan, W. H. Curtiss, D. G. Davison, J. P. Denby, C. O. Donaldson, George C. Eckley, F. H. Farmer, James G. Fish, W. R. French, V. E. Frizzell, W. W. Giles, T. E. A. Giller, R. H. Greer, W. C. Hanawalt, J. W. Haxby, J. Henderson, E. Herring, B. O. Johnson, C. H. Johnson, P. Justice, J. L. Kearney, D. O. Knisely, L. L. Knoble, A. H. Kyle, M. F. Leffingwell, E. P. Leresche, W. B. Lewin, E. J. List, E. G. Marten, C. B. McClelland, S. McCluer, E. J. McLeod, R. H. McMullen, Emil Mueller, S. E. St. John Paulet, J. S. Potter, J. Robards, J. Robertson, C. Schmitt, J. J. Schmitz, H. E. Sherman, T. F. Stanton, F. Tefft, G. E. Uehren, J. T. Unertl, E. J. Walter, W. H. Welch, M. B. Whitcomb, W. S. Winget, A. Youngberg.

The following are the class officers: A. E. Behnke, President; W. B. Lewin, Vice-President; D. O. Knisely, Secretary; J. Henderson, Treasurer; F. F. Brown, Valedictorian.

